# Targeting Cytokine Signaling and Lymphocyte Traffic via Small Molecules in Inflammatory Bowel Disease: JAK Inhibitors and S1PR Agonists

**DOI:** 10.3389/fphar.2019.00212

**Published:** 2019-03-13

**Authors:** Tamara Pérez-Jeldres, Christopher J. Tyler, Joshua D. Boyer, Thangaraj Karuppuchamy, Andrés Yarur, Daniel A. Giles, Shaila Yeasmin, Luke Lundborg, William J. Sandborn, Derek R. Patel, Jesús Rivera-Nieves

**Affiliations:** ^1^Inflammatory Bowel Disease Center, Division of Gastroenterology, University of California, San Diego, La Jolla, CA, United States; ^2^Department of Medicine, Pontifical Universidad Católica de Chile, Santiago, Chile; ^3^San Borja Arriarán Clinic Hospital, Santiago, Chile; ^4^VA San Diego Healthcare System, San Diego, CA, United States; ^5^Division of Gastroenterology and Hepatology, Medical College of Wisconsin, Milwaukee, WI, United States; ^6^La Jolla Institute for Allergy and Immunology, San Diego, CA, United States

**Keywords:** IBD, small molecules, JAK inhibitors, S1P agonists, MOA

## Abstract

The inflammatory Bowel diseases (IBDs) are a chronic, relapsing inflammatory diseases of the gastrointestinal tract with heterogeneous behavior and prognosis. The introduction of biological therapies including anti-TNF, anti-IL-12/23, and anti-integrins, has revolutionized the treatment of IBD, but these drugs are not universally effective. Due to the complex molecular structures of biologics, they are uniformly immunogenic. New discoveries concerning the underlying mechanisms involved in the pathogenesis of IBD have allowed for progress in the development of new treatment options. The advantage of small molecules (SMs) over biological therapies includes their lack of immunogenicity, short half-life, oral administration, and low manufacturing cost. Among these, the Janus Kinases (JAKs) inhibition has emerged as a novel strategy to modulate downstream cytokine signaling during immune-mediated diseases. These drugs target various cytokine signaling pathways that participate in the pathogenesis of IBD. Tofacitinib, a JAK inhibitor targeting predominantly JAK1 and JAK3, has been approved for the treatment of ulcerative colitis (UC), and there are other specific JAK inhibitors under development that may be effective in Crohn’s. Similarly, the traffic of lymphocytes can now be targeted by another SM. Sphingosine-1-phosphate receptor (S1PR) agonism is a novel strategy that acts, in part, by interfering with lymphocyte recirculation, through blockade of lymphocyte egress from lymph nodes. S1PR agonists are being studied in IBD and other immune-mediated disorders. This review will focus on SM drugs approved and under development, including JAK inhibitors (tofacitinib, filgotinib, upadacitinib, peficitinib) and S1PR agonists (KRP-203, fingolimod, ozanimod, etrasimod, amiselimod), and their mechanism of action.

## Introduction

Inflammatory Bowel diseases (IBDs) is a chronic immune-mediated condition of the gastrointestinal tract (Abraham and Cho, 2009). It is potentially caused by a dysregulated mucosal immune response to intestinal microflora in genetically predisposed hosts (Abraham and Cho, 2009). There are currently no curative therapies, and in most cases, lifelong treatment is required (Abraham and Cho, 2009). Non-specific immunomodulatory drugs such as glucocorticoids, sulfasalazine/5-aminosalicylates, methotrexate, and thiopurines were among the first drugs used to treat IBD (Soendergaard et al., 2018). The introduction of biologics during the last 20 years has revolutionized the treatment of IBD, and several anti-TNF monoclonal antibodies (mAbs) (including infliximab, adalimumab, certolizumab pegol, and golimumab) are commonly used. More recently, antibodies with a different mechanism of action (MOA), such as anti-integrin α4β7 (vedolizumab) and anti-IL12/IL23 (ustekinumab), became available for clinical use (Olivera et al., 2016). However, mAbs have limitations in terms of safety, cost, and sustained efficacy (Hemperly et al., 2018). In fact, around 10–30% of patients treated with anti-TNF are primary non-responders to therapy, and 23–46% are secondary non-responders (Hemperly et al., 2018). For these reasons, novel orally available drugs are still in great need and are being developed to treat IBD. The present review will focus on new families of chemically synthesized SM drugs already available or under development: Janus Kinases (JAK) inhibitors and sphingosine-1-phosphate receptor (S1PR) agonists, with emphasis on their MOA.

### Differences Between Small Molecules and Monoclonal Antibodies

Monoclonal antibodies are large molecules with high molecular weights (∼150 kDa) (Samanen, 2013). The mAb structure consists of four polypeptide chains, two identical heavy chains, and two identical light chains. Each mAb molecule has an antigen-binding region (Fab) or variable region, and a constant region or Fc (Ordás et al., 2012). The size and structure of the mAb determines the drug pharmacokinetic, target location, the drug–drug interaction, the antigenicity, and the route of administration. The mAbs are eliminated from the circulation by catabolism, which depends on the rates of proteolysis (extracellular degradation), recycling rates [by interaction with Brambell or the neonatal Fc receptor (FcRn)], and receptor-mediated antibody endocytosis rates (Ordás et al., 2012). Due to the large size of mAb the renal clearance is insignificant (Hemperly et al., 2018). Because of the protein composition of mAbs, the immune system can recognize them as immunogenic foreign antigens, which may lead to the development of specific anti-drug antibodies that nullify their therapeutic effect (Ordás et al., 2012; Yarur and Rubin, 2015; Hemperly et al., 2018). This results in increased drug clearance and ultimately may contribute to treatment failure and/or hypersensitivity reactions (Ordás et al., 2012; Yarur and Rubin, 2015; Hemperly et al., 2018). The addition of immunomodulators can decrease anti-drug antibody formation but increases the risks associated with inmunosupression (Ordás et al., 2012; Yarur and Rubin, 2015; Hemperly et al., 2018).

The term SM typically refers to organic compounds with low molecular weights, usually <1 kDa, which enables them to diffuse easily through cell membranes to reach intracellular targets (Samanen, 2013; Murphy and Zheng, 2015). Many SM inhibitors can function as immunomodulators due to their ability to specifically block intracellular signaling pathways thought to be pivotal to the pathogenesis of IBD (Samanen, 2013; Murphy and Zheng, 2015; Olivera et al., 2016). SMs have several advantages over conventional immunotherapeutic agents, including ease of administration (oral, without infusion costs), stable structures, non-immunogenic, potentially short half-lives, and usually lower manufacturing costs (Samanen, 2013; Murphy and Zheng, 2015; Olivera et al., 2016). Table 1 compares the main differences between SM and mAb (Samanen, 2013).

**Table 1:** Comparison of properties of SM drugs and mAbs (Samanen, 2013).

	Small molecules	Monoclonal antibodies
Molecular weight	Low (<1000 Da)	High (>1000 Da)
Preparation	Chemical synthesis	Biologically produced
Structure	Small organic compounds	Proteins
Route of administration	Oral	Parenteral
Location of target	Intracellular	Extracellular
Distribution	Variable in organs/tissues/cells	Limited to plasma and/or extracellular fluids
Metabolism	Metabolized typically by liver and gut CYPs into no active and active metabolites	Catabolism by proteolytic degradation to peptides and amino acids
Clearance	The clearance can be by renal excretion, biliary excretion, hepatic metabolism, and intestinal transporters	Mainly involves the reticuloendothelial system (RES) through proteolytic catabolism
Toxicity	Can produce specific toxicity due to parent or metabolites (often “off the target”)	Receptor-mediated toxicity
Antigenicity–hypersensitivity	No antigenic, but can show unpredictable hypersensitivity	Potential
Drug–drug interaction	Pharmacokinetic interactions by competitive clearance mechanism as: –Decreasing clearance by enzyme inhibition –Increasing clearance by enzyme induction	Infrequent
Mechanism of action	Receptor or enzyme inhibition	Depletion

### JAK-STAT Pathway and JAK Inhibitors

Cytokines are released by the immune system in response to a stimulus (Abbas et al., 2014b). They bind to specific receptors, triggering activation and initiation of intracellular signaling pathways (Abbas et al., 2014b). Cytokines encompass many structurally unrelated proteins that are grouped based on their binding to distinct receptor super families, which include Type I cytokine receptors, Type II cytokine receptors, the TNF receptor family, the IL-1 receptor family, and G-protein-coupled receptors. Each family of receptors utilizes different mechanisms of signal transduction (Table 2; Abbas et al., 2014b). The cytokines bind to the extracellular domain of the receptor, and trigger intracellular changes, resulting in signal transduction that drives changes in gene expression (Clark et al., 2013; Abbas et al., 2014b). Protein kinases have an essential role in the signal transduction pathway of these receptors, and are an attractive target to regulate the inflammatory response (Clark et al., 2013; Abbas et al., 2014b). However, due to the complexity and redundancy inherent to signal transduction networks, some of these kinases may be better therapeutic targets than others (Clark et al., 2013).

**Table 2:** Cytokines, receptors, and transduction pathway.

Ligands	Cytokine receptor	Transduction pathway	Function
	Type I		
Epo, Tpo, G-CSF, GH, and PRL	Homodimer receptor	JAK-STAT (JAK2)	Erythropoiesis Myelopoiesis Megakaryocyte/platelet production Growth Mammary development
IL-3, IL-5, and GM-CSF	Common β chain	JAK-STAT (JAK2)	
IL-6, IL-11, IL-23, and OSM	gp-130	JAK-STAT (mainly JAK1 but also JAK2, TYK2)	Naive T cells differentiation T-cell homeostasis Inflammation Granulopoiesis
IL-2, IL-4, IL-7, IL-9, IL-13, IL-15, and IL-21	Common γ chain	JAK-STAT (JAK1, JAK3)	Growth/maturation lymphoid cells Differentiation/homeostasis T cells, NK cells B cells class switching Inflammation
IFNα,IFNβ, IFNγ, IL-10, and IL-22	Type II	JAK-STAT (JAK1, JAK2, TYK2)	Antiviral Inflammation Antitumor
TNFα, TNFβ, LT, CD40, FasL, BAFF, April, Ox40, GITR, nerve growth factor	TNF receptor family	TRAF	Inflammation
IL-1, IL-18, IL-33	IL-1 receptor family	IRAK	Inflammation
Chemokines	Seven transmembrane G-protein-coupled receptors	G proteins	Chemotaxis and lymphocyte migration

*Epo, erythropoietin; Tpo, thrombopoietin; G-CSF, granulocyte-colony stimulating factor; GH, growth hormone; PRL, prolactin; IL, interleukin; GM-CSF, granulocyte–macrophage colony-stimulating factor; OSM, oncostatin M; TNF, tumor necrosis factor; LT, lymphotoxin; FasL, Fas ligand, B-cell activating factor; GITR, glucocorticoid-induced TNF receptor; JAK, Janus kinase; STAT, signal transducers and activators of transcription; TRAF, TNF receptor-associated factors; IRAK, interleukin-1 receptor-associated kinases (Abbas et al., 2014d).*

The JAK family is a small family of receptor-associated tyrosine kinases that are essential for the cytokine signaling cascade, downstream of Type I and Type II cytokine receptors (Schwartz et al., 2017). The JAK-signal transducers and activators of transcription (STAT) pathway plays an important role in innate immunity, adaptive immunity, and hematopoiesis, participating in cellular processes such as cell growth, survival, differentiation, and migration (Table 2; Banerjee et al., 2017; Olivera et al., 2017). There are four members of the JAK family (JAK1, JAK2, JAK3, and TYK2) and seven signal transducers and transcription activators called signal transducer and activator of transcription, or STAT (STAT 1–4, 5a, 5b, and 6) (Clark et al., 2013; Banerjee et al., 2017; Olivera et al., 2017; Schwartz et al., 2017; Table 3).

**Table 3:** STAT and cellular function.

STAT	Cellular function
1	Cell growth and apoptosis TH1 cell-specific cytokine production Antimicrobial defense
2	Mediation of IFNα/IFNβ signaling
3	Cell proliferation and survival Inflammation Immune response Embryonic development Cell motility
4	TH1 cell differentiation Inflammatory responses Cell proliferation
5A	Cell proliferation and survival IL-2Ra expression in T lymphocytes Mammary gland development Lactogenic signaling
5B	Cell proliferation and survival IL-2Ra expression in T lymphocytes Sexual dimorphism of body growth rate NK cell cytolytic activity
6	Inflammatory and allergic immune response B-cell and T-cell proliferation TH2 cell differentiation

*TH, T helper; IFN, interferon; IL, interleukin. Table adapted from Miklossy et al. (2013). Therapeutic modulators of STAT signaling for human diseases (Miklossy et al. 2014).*

The unique structure of each JAK clearly distinguishes them from other members of the protein tyrosine kinase family (Banerjee et al., 2017). The JAKs contain four functional domains: the SH2 domain (a scaffold for STAT), the FERM domain (regulates catalytic activity and mediates association with receptors and other proteins), the pseudo-tyrosine kinase domain, and a catalytically active tyrosine kinase domain (Banerjee et al., 2017). These last two domains are the basis for the name of the protein family named Janus (the two-faced Roman god of beginnings, endings, and duality), thus JAK exhibits a domain with kinase activity, while the other negatively regulates the activity of the first (Banerjee et al., 2017).

**FIGURE 1:**
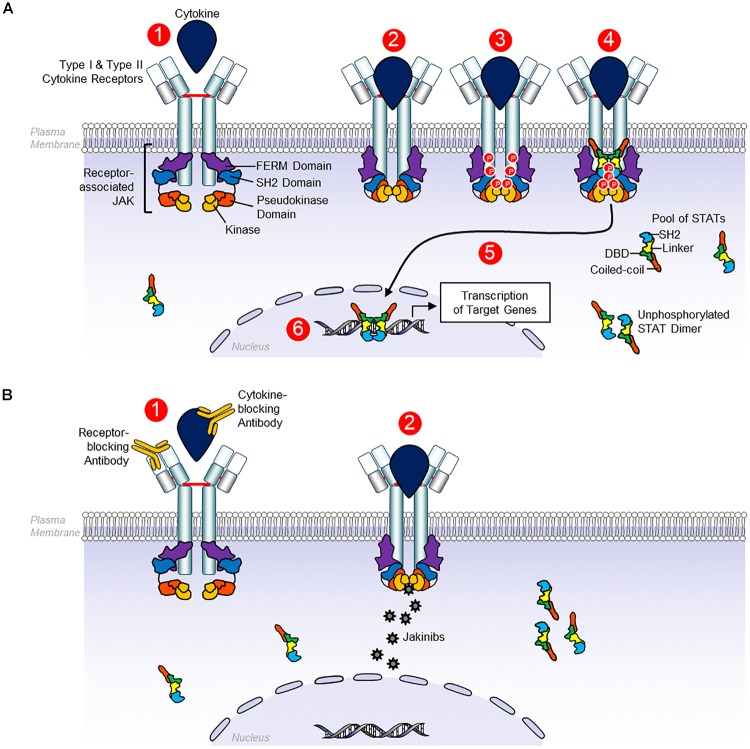
Signaling by receptors Type I and Type II cytokines. **(A)** Type I and type II cytokine receptors comprise subunits that physically associate with Janus kinases (JAKs). Type I and type II cytokine depend on JAKs to transduce intracellular signals. JAK proteins share four components: the kinase domain, the pseudokinase domain, the FERM domain, and the SH2 domain. 1, the canonical JAK-STAT signaling begins with the extracellular association between cytokines and their corresponding transmembrane receptors. 2, the receptor dimerization triggers the transactivation of JAK. 3. Phosphorylation of the cytoplasmic tails of the receptors that create docking sites for STATs. 4–6, STAT binds to JAK, allowing the tyrosine phosphorylation of STAT which results in STAT dimerization, nuclear translocation, DNA binding, and ultimately, modulation of gene transcription. Unphosphorylated STAT dimers also have regulatory functions, although these functions are less well defined. **(B)** 1, monoclonal antibodies can block Type I and Type II cytokines and their receptors. 2, by contrast, JAK inhibitors block cytokine signaling, binding to the kinase domain of JAK in the ATP-binding site, avoiding their phosphorylation and JAK activation, preventing STAT phosphorylation and other substrates, so intracellular signals cannot be transduced (Schwartz et al., 2017).

Canonical JAK-STAT signaling starts with the binding between cytokines and their corresponding transmembrane receptors, allowing receptor dimerization and triggering the transactivation of JAK, followed by phosphorylation of the cytoplasmic tails of the receptors that produce coupling sites for STAT, resulting in the tyrosine-phosphorylation (p-Tyr) of the STAT by JAK (Jatiani et al., 2010; Villarino et al., 2017). After these events, STAT (like homo/heterodimers) translocate to the nucleus, bind to DNA, and modulate gene transcription (Jatiani et al., 2010; Villarino et al., 2017). In addition to STAT phosphorylation, other kinases such as Src, phosphoinositide 3-kinases (PI3K), and RAF can be phosphorylated, activating additional signaling pathways involving proteins including Akt, and extracellular signal-regulated kinases (ERK) (Figure 1; Jatiani et al., 2010).

Signal transducers and activators of transcription is under the control of physiological negative regulators such as (i) suppressors of cytokine signaling (SOCS), that inhibit the kinase activity, binding phospho-tyrosine residues and competing with STAT at cytoplasmic level, (ii) protein tyrosine phosphatases (PTPs) that inactivate JAK and STAT in both the nucleus and the cytoplasm, (iii) protein inhibitor of activated STAT family (PIAS) that interferes at the nuclear level with STAT-mediated transcription and triggers proteasome degradation, and (iv) the modulators SH2B adaptor protein that increase or decrease JAK activation (Villarino et al., 2017).

Many cytokines implicated in the pathogenesis of immune-mediated diseases use the JAK-STAT pathway, representing a potential therapeutic target for these disorders (Jatiani et al., 2010; Clark et al., 2013; Banerjee et al., 2017; Olivera et al., 2017; Schwartz et al., 2017; Villarino et al., 2017). The mAbs can block Type I and Type II cytokines and their receptors. By contrast, JAK inhibitors block cytokine signaling, binding to kinase domain of JAK at the ATP-binding site, avoiding their phosphorylation and JAK activation, preventing STAT phosphorylation and other substrates, so intracellular signals cannot be transduced (Jatiani et al., 2010; Clark et al., 2013; Banerjee et al., 2017; Olivera et al., 2017; Schwartz et al., 2017; Villarino et al., 2017). Other potential therapeutic candidates include STAT-binding inhibitory peptides, STAT inhibitors, STAT-targeting small interfering RNA (siRNA), and STAT-binding decoy oligonucleotides (Schwartz et al., 2017; Villarino et al., 2017).

The JAK inhibitors have been used in the treatment of hematologic disorders (Jatiani et al., 2010). In recent years, these inhibitors have received attention for the treatment of autoimmune/immune-mediated disorders such as rheumatoid arthritis (RA) (Vanhoutte et al., 2017), systemic lupus erythematosus (SLE) (ClinicalTrials.gov, ClinicalTrials, 2018e), dermatomyositis (Hornung et al., 2014), Sjogren syndrome (ClinicalTrials.gov, ClinicalTrials, 2018n), vasculitis (Zhang et al., 2018), psoriasis (Hsu and Armstrong, 2014), alopecia areata (Divito and Kupper, 2014), atopic dermatitis (Levy et al., 2015), vitiligo (Liu et al., 2017), and IBD (Panés et al., 2017; Sandborn et al., 2017a; Vermeire et al., 2017a).

### JAK Inhibitors for the Treatment of IBD

Typically, IBD is associated with chronic inflammation, defined by a dysregulated response of the innate and adaptive immune systems (Abraham and Cho, 2009; Boland and Vermeire, 2017). Chronic inflammation in Crohn’s disease (CD) is characterized by a response of helper T cells type 1 (Th1) and helper T cells type 17 (Th17), with inadequate activity of regulatory T cells (Treg), whereas UC has generally been considered a type 2 T helper cell cytokine profile (Th2) (Boland and Vermeire, 2017). In both diseases, many of the cytokines produced by these T cells signal through JAK receptors; therefore, JAK proteins have an important place in the signaling of inflammation in IBD (Boland and Vermeire, 2017).

Key cytokines in the pathogenesis of IBD belong to Type I and Type II cytokines receptors [i.e., IL-6, IL-5, IL-9, IL-10, IL-13, IL-12/23, IL-22, granulocyte–macrophage colony-stimulating factor (GM-CSF), IFN-γ] (Zhou et al., 2007; Dienz and Rincon, 2009; Abbas et al., 2014a,c; Flamant et al., 2017). These cytokines all signal through the JAK/STAT pathway. In contrast, the cytokines TNF, IL-1, and IL-17, which are the major drivers of IBD, do not use the JAK-STAT pathway in their signaling pathways (Zhou et al., 2007; Dienz and Rincon, 2009; Abbas et al., 2014a,c; Flamant et al., 2017). However, these cytokines induce the expression of a wide range of downstream pro-inflammatory cytokines, that in turn depend on JAK/STAT signaling (Zhou et al., 2007; Dienz and Rincon, 2009; Abbas et al., 2014a,c; Flamant et al., 2017).

Interleukin-6 (IL-6) along with oncostatin M (OSM) and IL-11 signal through the gp130-associated receptor family. IL-6 activates JAK1, JAK2, and TYK2 leading to STAT3 transduction, which promotes T cell proliferation, favoring the polarization of Th17 cells (Zhou et al., 2007; Banerjee et al., 2017). Notably, IL-6 can also promote Th2 differentiation (Dienz and Rincon, 2009). In addition, IL-6 has other functions relevant to IBD, such as regulating intestinal permeability, by its effects on tight junctions, regulating the proliferation of epithelium, and healing of wounds (Flamant et al., 2017).

Interleukin-12 and IL-23 also play an important role in IBD, and JAK2 and tyrosine kinases type 2 (TYK2) are involved in the signaling of these cytokines by activating STAT3 and STAT4, promoting inflammatory reactions through their ability to induce Th1 and Th17 polarization, respectively, and production of IFN-γ, IL-21, and IL-22 (Flamant et al., 2017).

Interleukin-10 is an anti-inflammatory cytokine produced by many immune cell populations, including activated macrophages, dendritic cells, regulatory T cells, Th1 and Th2 cells. IL-10 activates JAK1 and TYK2 proteins, leading to STAT3 phosphorylation (Abbas et al., 2014a). The anti-inflammatory effects of IL-10 results, in part, from its ability to inhibit the production of IL-12 by activated macrophages and dendritic cells as well as inhibiting the expression of costimulatory and class II MHC molecules in these cells (Abbas et al., 2014a).

Interleukin-22 is produced in epithelial tissues, especially in the skin and gastrointestinal tract. IL-22 activates JAK1 and TYK2, transducing signals via STAT3, STAT1, and STAT5. IL-22 has a role in maintaining epithelial integrity, mainly by promoting the barrier function of epithelial cells and by inducing production of anti-microbial peptides (Abbas et al., 2014c). However, IL-22 contributes to inflammation, in part by stimulating epithelial production of chemokines, and may therefore be involved in tissue injury in inflammatory diseases (Abbas et al., 2014c).

Interleukin-9 binding to its receptor leads to activation of JAK1 and JAK3, which in turn phosphorylates STAT1/STAT3 and STAT1/STAT5, respectively (Flamant et al., 2017). IL-9 has been associated with deleterious impact on intestinal epithelial wound healing (Flamant et al., 2017).

Interferon-γ activates JAK1 and JAK2, inducing STAT1 activation, resulting in macrophage activation, Th1 polarization, and increased expression of several proinflammatory cytokines. However, IFN-γ also has a protective function in epithelial healing (Flamant et al., 2017). Moreover, IFN-γ protects from tissue destruction by inhibiting the expression of genes that code for tissue destructive factors such as matrix metalloproteinases (MMPs), serine proteases, coagulation factors, complement components, and enzymes involved in the metabolism of prostaglandin. In addition, IFN-γ decreases neutrophil and monocytes infiltration (Hu and Ivashkiv, 2009). GM-CSF activates JAK2 which phosphorylates STAT5, and STAT3 promoting monocyte/macrophage/granulocyte survival and activation (Kimura et al., 2009; Flamant et al., 2017).

Drugs that block JAK/STAT signaling have the potential to alter multiple inflammatory pathways, being less specific in their action than drugs that target specific cytokines or their receptors (Soendergaard et al., 2018). This complexity is clear for IL-6 (pro-inflammatory) and IL-10 (anti-inflammatory) signaling, where both ligands, despite activating JAK1 and STAT3, have opposing functions (Soendergaard et al., 2018). Consequently, blocking JAK1 affects both IL-6 and IL-10, and may alter the inflammatory balance in both directions (Soendergaard et al., 2018). Additionally, JAK inhibitors can result in undesirable adverse effects like cytopenia and infectious complications, through its blockade of GM-CSF and IFN-γ signaling, respectively (Clark et al., 2013). On the other hand, a major strength is their effectiveness. Through adequate plasma levels, these drugs induce partial and reversible inhibition of cytokine signaling, resulting in a better balance between the inflammatory and immunomodulatory response (Clark et al., 2013). More selective inhibition of the JAK-STAT pathway is being developed and may overcome the challenges of less selective inhibitors.

The US Food and Drug Administration (FDA), in May 2018, approved tofacitinib as the first JAK inhibitor to treat moderate severely active UC (Soendergaard et al., 2018). Similar to the FDA, the Committee for Medicinal Products for Human Use (CHMP) at the European Medicines Agency (EMA) had a favorable opinion, and recommended their use in adult patients with moderately to severely active UC with inadequate or loss of response or intolerance to either conventional therapy or biological agents. Currently, no JAK inhibitors are approved for CD; however, other selective JAK inhibitors are in the pipeline for CD (Soendergaard et al., 2018).

### Tofacitinib

Tofacitinib (Xeljanz, Pfizer) is a pan-JAK inhibitor, that preferentially inhibits JAK1 and JAK3, in a dose-dependent fashion (Sandborn et al., 2017a). Tofacitinib has a predicted gut availability of 93%, and the clearance is 70% hepatic, whereas the remaining 30% is cleared by renal metabolism (Hemperly et al., 2018). Tofacitinib’s half-life is 3 h and neither age, gender, body weight, or disease severity at baseline have an effect on its clearance or plasma levels (Dowty et al., 2014).

A double-blind, placebo-controlled phase 2 study evaluated the efficacy of tofacitinib in patients with UC (*n* = 194) with moderate to severe activity (Sandborn et al., 2012). The patients were randomly assigned during 8 weeks to different tofacitinib doses (0.5, 3, 10, and 15 mg each 12 h) or placebo. The primary outcome at 8 weeks (clinical response established as the decrease of at least three points and at least 30% from the baseline total Mayo score, and decrease of at least one point or an absolute rectal bleeding sub-score of 0 or 1) reported a statistically significant response between the higher doses versus placebo (78% versus 42%, respectively) (Sandborn et al., 2012). These data were supported by phase 3, double-blind placebo-controlled studies; OCTAVE induction 1, 2, and OCTAVE sustain. In the induction trials; OCTAVE 1 (*n* = 598) and 2 (*n* = 591) trials, the patients were randomly assigned to receive 10 mg of tofacitinib twice daily or placebo during 8 weeks (Sandborn et al., 2017a). The primary endpoint was remission at week 8 (a total Mayo score of ≤2, with no subscore > 1 and a rectal bleeding sub-score of 0). This endpoint was achieved in 18.5% in the tofacitinib-treated group versus 8.2% in the placebo group (*P* = 0.007); in the OCTAVE Induction 2 trial, remission was achieved in 16.6% versus 3.6% (*P* < 0.001). A total of 593 patients achieved clinical response after the induction therapy and were recruited in the OCTAVE Sustain trial to randomly receive tofacitinib as maintenance therapy (5 or 10 mg twice daily) or placebo during 52 weeks. The aim endpoint (remission at 52 week) was achieved in 34.3 and 40.6% (5 and 10 mg twice daily, respectively) versus 11.1% placebo (*P* < 0.001) (Sandborn et al., 2017a). Furthermore, mucosal healing was more frequent in the tofacitinib group, and tofacitinib was effective in both treated and naïve to anti-TNF patients. The safety and efficacy data were evaluated in a phase 3, multicenter, open-label, long-term extension study in patients with severe to moderate UC (*n* = 946). Preliminary data showed that no new safety concerns emerged, compared with those observed in RA. Efficacy results from OLE study (NCT01470612) support sustained efficacy with tofacitinib at both 5 and 10 mg doses twice daily (Lichtenstein et al., 2017).

Similar studies were conducted in patients with moderate to severe CD; In a phase II (*n* = 139) study, patients were randomly assigned to receive tofacitinib (1, 5, or 15 mg twice daily) or placebo during 4 weeks. This study did not show a significant clinical response or remission response (Sandborn et al., 2014). Subsequently, another phase IIb study was performed. In this study, patients were randomized, during 8 induction weeks, to tofacitinib 5 mg twice per day (*n* = 86) or placebo (*n* = 91). The responders were included in the maintenance phase, during 26 weeks, to receive tofacitinib 5 or 10 mg daily or placebo. The majority of enrolled patients were previously treated with anti-TNF (76–79%). In this study, the results were also disappointing, despite the long duration of treatment, the remission rates did not reach significant differences (Panés et al., 2017).

These discouraging results in CD may be due to high placebo response rates or differences in the fundamental immunopathogenesis of CD and UC. Several factors may have contributed to the high placebo response observed, including lack of centralized reading endoscopy and absence of baseline objective markers of disease activity (Panés et al., 2017).

**Filgotinib** (GLPG0634, Galapagos/Gilead Sciences) is an oral JAK1 inhibitor, with enhanced selectivity for JAK1 over JAK2 and JAK3 (30 and 50 times, respectively) in blood (Vermeire et al., 2017a,b; Hemperly et al., 2018). Filgotinib dosing leads to the formation of active metabolite which exhibits a similar JAK1 selectivity profile as the parent compound, but has less potency (Vermeire et al., 2017a,b; Hemperly et al., 2018). Still, both contribute to the clinical activity of filgotinib. The half-life of filgotinib is 6 h, while the metabolite has a terminal elimination half-life of 21–27 h. Filgotinib and its metabolites are predominantly cleared renally (>80%) (Vermeire et al., 2017a,b; Hemperly et al., 2018).

FITZROY, a double-blind, placebo-controlled study, examined the efficacy and safety of filgotinib for the treatment of active moderate to severe CD (Vermeire et al., 2017a). A total of 174 patients with active CD were enrolled. Disease activity was confirmed by centrally read endoscopy. A proportion of patients achieved clinical remission with filgotinib 200 mg once a day, compared with placebo (47 versus 23%; *p* = 0.077) at week 10. Data also suggested that filgotinib is effective in anti-TNF exposed and naïve patients, being twofold higher in TNF-naïve group (Vermeire et al., 2017a). In addition, a recent *post hoc* analysis showed that clinical remission is still seen in CD, regardless of the disease location or duration (Vermeire et al., 2017b). Currently, there are phase III trials underway in both a CD and UC (ClinicalTrials.gov, ClinicalTrials, 2018h,i,j,m).

**Peficitinib** (GLPG1205, Janssen) is JAK1 and JAK3 inhibitor (Sands et al., 2018; Soendergaard et al., 2018). The efficacy and safety of the drug has been evaluated for the treatment of moderate to severe UC (*n* = 219) in a multicenter, randomized, double blind, placebo-controlled, phase IIb trial. Nevertheless, the development of this drug was discontinued in 2017 due to disappointing efficacy results (Sands et al., 2018; Soendergaard et al., 2018).

**Upadacitinib** (UPA) (ABT-494, Abbvie) is a JAK1-selective inhibitor. It is a non-sensitive substrate for cytochrome P450, approximately 20% is eliminated, unchanged, in urine (Hemperly et al., 2018). Its efficacy and safety were assessed in patients with moderate-to-severe CD who had inadequate response, or intolerance, to anti-TNF (Sandborn et al., 2017b). In this study, patients receiving 6 mg twice daily (27%) achieved clinical remission at a higher rate than placebo (11%). A significant dose–response relationship for endoscopic remission was observed in the UPA arm (Sandborn et al., 2017b). In addition, patients with moderate-to-severely active UC (*n* = 250), and history of inadequate response, loss of response or intolerance to corticosteroids, immunosuppressant, or biologic therapies, were included in a phase IIb double-blind placebo-controlled dose-ranging induction study, to assess the safety and efficacy of UPA. At week 8, both the primary objective: clinical remission per Adapted Mayo Score (stool frequency subscore ≤ 1, rectal bleeding score = 0, endoscopic score ≤ 1) and the secondary objectives: clinical remission per full Mayo score, clinical response per adapted Mayo score and endoscopic improvement were evaluated (Sandborn et al., 2018b). All of these objectives were achieved with different doses ranging from 15 to 45 mg QD. The tolerance to UPA was good and safety was similar to that of other UPA studies (Sandborn et al., 2018b). Phase III studies in CD and UC are ongoing (ClinicalTrials.gov, ClinicalTrials, 2018g,l,o,p).

**TD-1473** (Theravance, Biopharma) is a new oral pan-JAK inhibitor being investigated (ClinicalTrials.gov, ClinicalTrials, 2018f; Soendergaard et al., 2018). Unlike other JAK inhibitors, its distribution is limited to the gastrointestinal tract, minimizing systemic toxicity and side effects (ClinicalTrials.gov, ClinicalTrials, 2018f; Soendergaard et al., 2018). Data from phase I study in healthy volunteers have shown that treatment with TD-1473 is safe and well-tolerated (Beattie et al., 2018). The safety, tolerability, and pharmacokinetics of TD-1473 were assessing in a double-blind placebo-controlled multicenter phase Ib study in subjects with moderately to severely active UC (*n* = 40) (ClinicalTrials.gov, ClinicalTrials, 2018f; Sandborn et al., 2018a). In this study, TD-1473 was generally well tolerated over 4 weeks with evidence of intestinal restriction, low systemic exposure, and signals for clinical and biomarker activity in subjects with moderately to severely active UC (ClinicalTrials.gov, ClinicalTrials, 2018f; Sandborn et al., 2018a).

**Pf-06651600/Pfizer** (JAK3 inhibitor) and **Pf-06700841/Pfizer** (TYK2/JAK1 inhibitor) are being tested in clinical trials to be completed by early 2020 (ClinicalTrials.gov, ClinicalTrials, 2018k,q).

#### Adverse Effects: Experience From IBD and Rheumatoid Arthritis

Most of the safety information currently for JAK inhibitors belongs to RA and psoriasis literature. For S1PR agonists most of the safety data originated from Multiple Sclerosis and IBD trials, as tofacitinib and fingolimod were approved for those applications years earlier. Otherwise, post-marketing real-world data from clinical practice after the approval of tofacitinib in immune-mediated disease as RA and IBD are available (Hsu and Armstrong, 2014; Charles-Schoeman et al., 2015; Liu et al., 2017; Schwartz et al., 2017; Winthrop, 2017; Cohen et al., 2018; Kang et al., 2018; Verden et al., 2018).

Tofacitinib is the JAK inhibitor whose side effects are best known compared to other more specific JAK inhibitors. Still is unknown if the higher selectivity of the new JAK will result in fewer adverse effects (Winthrop, 2017).

### Infections

The risk of serious infections during JAK inhibitor treatments is similar to that of biologics and most infections do not require treatment discontinuation. Nasopharyngitis and influenza are the most frequently reported infection-related adverse events. Tuberculosis and osteomyelitis are infrequent infections that also have been identified, and in this circumstance, the therapy must be interrupted (Winthrop, 2017). In addition, JAK inhibitors increase the risk of herpes zoster infection. However, Shingrix (recombinant zoster vaccine, GlaxoSmithKline) can reduce risk of infection and associated complications in patients treated with JAK inhibitors (Winthrop, 2017).

Other serious viral infections like nephropathy by BK virus (a polyoma virus) have been identified with the use of tofacitinib during renal trasplantation (Schwartz et al., 2017). A few cases of cytomegalovirus (CMV) infections, including CMV retinitis, have occurred in patients under treatment with tofacitinib in the long-term extension studies (Sandborn et al., 2014; Schwartz et al., 2017; Winthrop, 2017). Also cases of abscesses, cellulitis, *Clostridium difficile* infection, pneumonia by *Pneumocystis jiroveci*, candida infections, urinary tract infections, and histoplasmosis have been reported (Sandborn et al., 2014).

### Malignancy

All immunosuppressants have the potential to increase cancer risk. Accordingly, JAK inhibitors could interfere with T and NK immune vigilance against cancer and the antineoplastic role of IFN-γ (Schwartz et al., 2017).

Recently, the post marketing surveillance (PMS) data of worldwide tofacitinib use in RA, obtained from Pfizer safety database during a 3-year reporting period, was published. The estimated exposure to tofacitinib was 34,223 patient years. The overall relative risk was 0.45 per 100 patients-year, being highest during the first year and stabilizing later. The most notified neoplasms were lymphoma, skin, lung, breast, brain, prostate, uterine and colon cancer, malignant melanoma, squamous, and basal cell carcinoma. During the PMS, the most reported cancer in RA patients receiving tofacitinib therapy was the non-melanoma skin cancer (NMSC) (Cohen et al., 2018).

### Dyslipidemia and Cardiovascular Events

A dose-dependent increase in HDL, LDL, and total cholesterol has been observed. Levels normalized after cessation of treatment. This change in lipid profiles has not been found to be associated with an increase of adverse cardiovascular events (Charles-Schoeman et al., 2015).

Thromboembolic events were reported during a placebo-controlled trial of baricitinib, a JAK inhibitor tested in RA. A post-marketing adverse event report from the FDA’s Adverse Event Reporting System did not show increased risk of thromboembolic events for tofacitinib, tofacitinib extended-release, or ruxolitinib. However, the data indicated that pulmonary thrombosis and portal vein thrombosis may be a class-wide risk for JAK inhibitors (Kang et al., 2018; Verden et al., 2018).

### Anemia and Leukopenia

Because hematopoietic growth factors signal through JAK2, cytopenia is frequent with the use of the first-generation pan-JAK inhibitors. These alterations are usually well tolerated and do not require treatment discontinuation (Schwartz et al., 2017).

### Pregnancy

There is a lack of information about the effect of JAK inhibitors during pregnancy since most studies exclude pregnant women, and there is little data available from patients who became pregnant while receiving the medication (Winthrop, 2017). The pregnancy results from patients with UC under tofacitinib exposure were reported. Mahadevan et al. (2018) notified that from 1157 UC patients recruited in interventional trials, 25 cases were reported (11 maternal, 14 paternal) with exposure to tofacitinib. These results include 15 healthy neonates, 2 spontaneous abortions, and 2 medical interruptions. Cases of fetal death, neonate death, and congenital malformations were not described.

The data available to date does not allow to definitive position regarding the tofacitinib effect on pregnancy, and its use is not recommended (Mahadevan et al., 2018).

### Others Adverse Events

Intestinal perforation and elevated serum liver enzymes have been reported with the use of tofacitinib (Olivera et al., 2016).

### Future Perspectives

The pathogenesis of UC and CD involve different signaling pathways, which may explain the differential response to diverse drugs. The use of a drug with different MOA could be an effective alternative (i.e., tofacitinib for anti-TNF non-responders in UC). Further understanding the main pathways involved in the pathogenesis of IBD may predict the efficacy of specific drugs based on their MOA in the near future (Jabeen et al., 2015).

Janus kinases inhibitors target a broad spectrum of cytokines, and are a safe and effective treatment for immune-mediated disorders, such as IBD. As stated previously the JAK-STAT pathway plays an important role in innate and adaptive immunity, cell growth, survival, differentiation, and migration; hence, there are concerns of potential off-target effects. However, the safety profile to date is similar to other biological agents (Winthrop, 2017). Selectivity of the new JAK inhibitors may improve safety, while maintaining efficacy. The development of drugs such as TD-1473, with action limited to the gastrointestinal tract and less systemic exposure, may also improve safety.

In cases of refractory illness, an emergent idea is the combined use of drugs that target distinct pathways, such as inhibitors of kinase PI3K or receptor tyrosine kinases (RTKs) (Montor et al., 2018).

Signal transducers and activators of transcriptions do not have intrinsic catalytic activity unlike JAK and RTKs, whose kinase domains are an obvious therapeutic target. A potential and attractive approach is the inhibition of STAT using oligonucleotides, which would sequester STAT away from the nucleus. Small molecules (SMs), inhibitory peptides, and siRNAs that target STATs are also undergoing clinical trials for other diseases (Villarino et al., 2015; ClinicalTrials.gov, ClinicalTrials, 2018b).

The relative risk and benefits of these drugs as monotherapy, combination, or sequential therapy with other drugs remain incompletely characterized (Barroso et al., 2017).

### S1P/S1PR Targeting

Sphingosine-1-phosphate (S1P) is a sphingosine-derived phospholipid that binds to 5 G-protein-coupled receptors (S1PR1-5) (Park and Im, 2017). The S1P receptors are involved in several physiological events and cellular processes, such as adhesion, migration, lymphocyte/hematopoietic cell trafficking, endocytosis, vascular tone and permeability, embryogenesis, angiogenesis, and cardiac function (Sanchez and Hla, 2004; Gonzalez-Cabrera et al., 2014).

**FIGURE 2:**
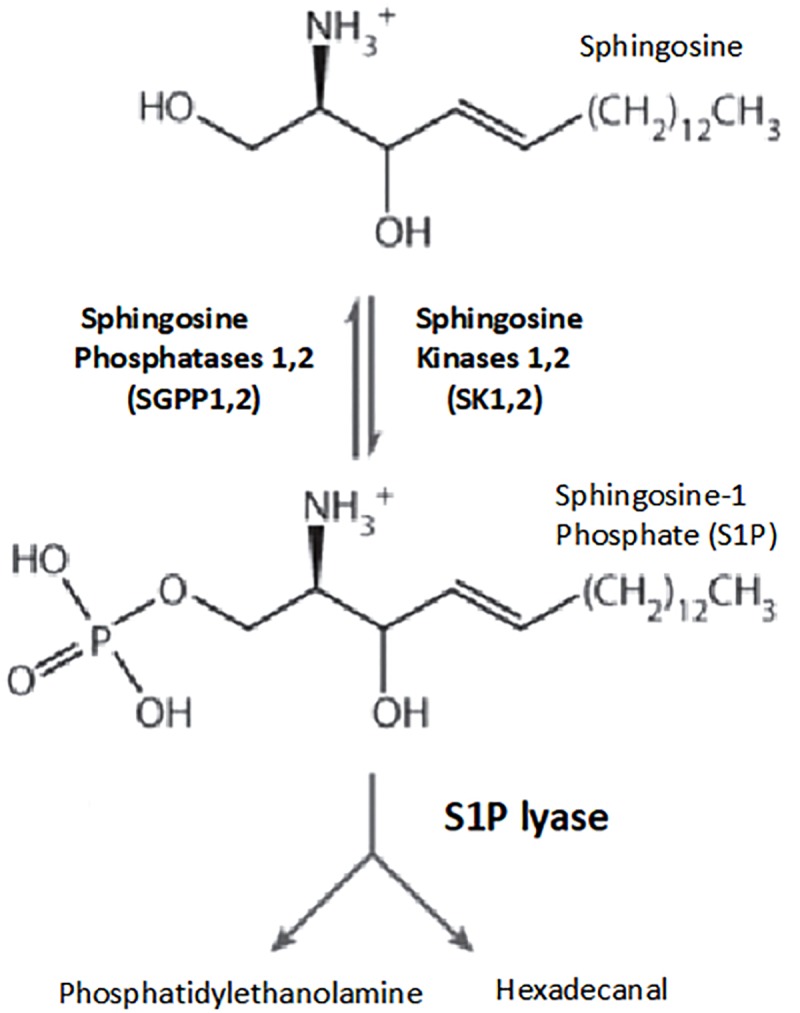
Pathways of sphingosine-1-phosphate metabolism. Key enzymes for the formation and degradation of S1P. S1P is produced by the phosphorylation of sphingosine by sphingosine kinase. S1P can then be metabolized by S1P lyase to phosphoethanolamine and hexadecanal, which are then further metabolized into glycerophospholipids and phosphatidylethanolamine, respectively. Conversely, S1P phosphohydrolase regenerates sphingosine by dephosphorylating S1P. SphK, sphingosine kinase; SPP, sphingosine-1-phosphate phosphohydrolase; SPL, sphingosine-1-phosphate lyase (Le Stunff et al., 2004).

Sphingolipids are important elements in the structure of cell membrane, and S1P is a sphingolipid metabolite derived from sphingosine. S1P is phosphorylated by sphingosine kinases 1 and 2, and reversibly dephosphorylated by sphingosine phosphatases 1,2 and by the action of S1P lyase, S1P is irreversibly degraded (Le Stunff et al., 2004; Figure 2).

Sphingosine-1-phosphate/S1PR1 interactions are relevant for lymphocyte trafficking through the thymus, secondary lymphoid organs, circulation, and tissues. S1P mediates the traffic of dendritic cells, B cells, and T cells (naive and central memory-CCR7-positive), but does not have a significant role in the chemotaxis of effector memory CCR7-negative T cells, which maintain tissue immune-surveillance (Abbas et al., 2014a; Perez-Jeldres et al., 2018). The S1P lyase distribution, higher in tissues but absent in the vasculature, favors an S1P concentration gradient between the blood (higher levels), lymph, secondary lymphoid organs, and tissues (lower levels), determining the movement from the areas with low concentration to high S1P concentration (Abbas et al., 2014a; Perez-Jeldres et al., 2018). Elevated S1P levels in blood induce S1PR1 internalization, whereas in the lymph node and tissues S1PR1 is re-expressed after some hours, and during this time the T cell is able to interact with antigen-presenting cells (Abbas et al., 2014a; Perez-Jeldres et al., 2018). Once S1PR1 re-appears on the surface of lymphocyte, these cells can leave the lymph node or tissue by sensing the higher S1P concentration in the blood, determining immune cell egress into the circulation (Horga and Montalban, 2008; Olivera et al., 2016).

### Mechanisms of Action of S1PR Modulators

**FIGURE 3:**
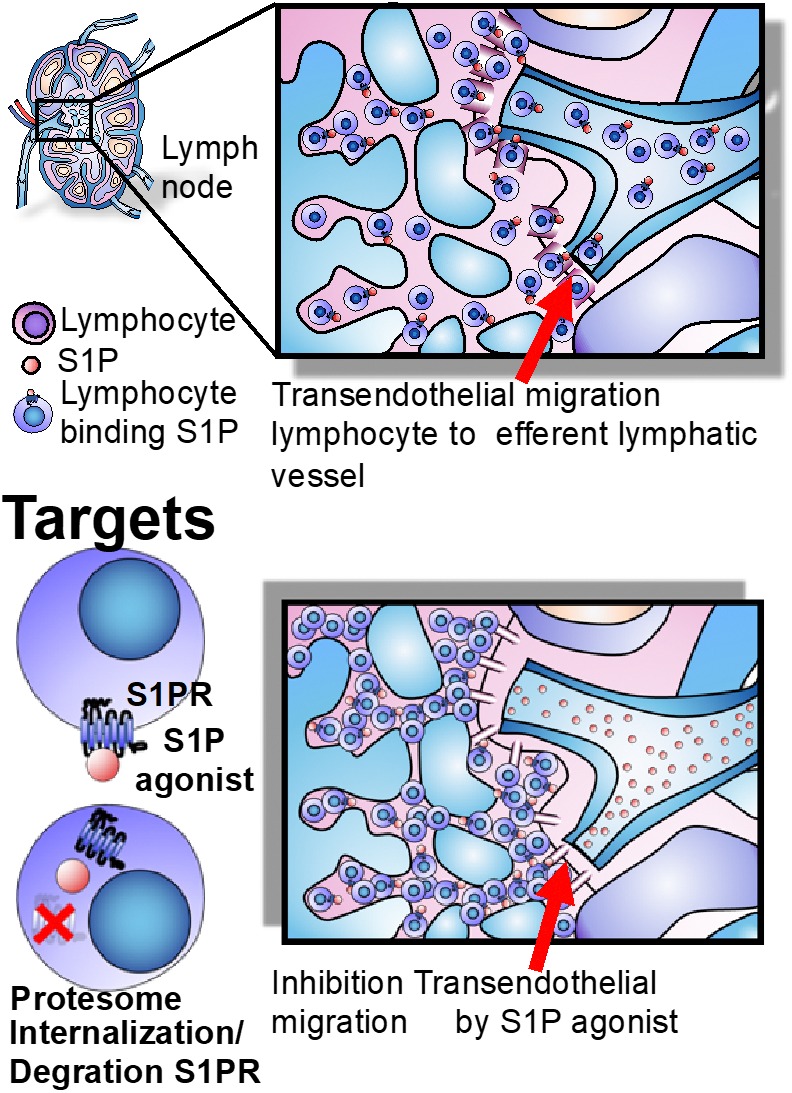
Lymph node egress and targets. The egress of lymphocytes from lymph nodes is dependent on the S1P gradient, whose concentration is higher in blood than lymph nodes and effector tissues. S1PR agonists induce long-lasting receptor downregulation and lymphocyte sequestration in lymphoid tissues and inhibit transendothelial migration of T cells across the lymphatic endothelial barrier in the lymph node, where they remain sequestered (Perez-Jeldres et al., 2018).

The native ligand S1P indices internalization of S1PR, which are recycled back to the cell surface within several hours, achieving a transitory lymphopenia (Park and Im, 2017). By contrast, S1PR1 agonists lead to the internalization of the receptor and subsequent ubiquitination and proteasome degradation of the receptor, producing sustained lymphopenia that renders lymphocytes incapable of following the S1P gradient and exiting the lymph node. This sequestration potentially prevents their access to sites of inflammation (Abbas et al., 2014a; Park and Im, 2017; Perez-Jeldres et al., 2018). In addition, S1PR1 is strongly expressed by lymphatic endothelium, where it tightens the lymphatic endothelial barrier. S1PR1 agonists can therefore interfere with lymphocyte trafficking by inhibiting transendothelial migration and blocking lymphocyte egress from the lymph node. These effects are reversed upon withdrawal of the agent (Horga and Montalban, 2008; Perez-Jeldres et al., 2018; Figure 3).

Sphingosine-1-phosphate signaling is involved in multiple immune functions. Therapies targeting the S1P axis may be applicable to treat autoimmune/immune-mediated diseases and have been tested in MS, RA, SLE, psoriasis, and IBD (Perez-Jeldres et al., 2018).

### S1PR Modulators in IBD

**Fingolimod/**FTY720 (Gilenya^TM^) is an S1P-analog, acting as non-selective potent agonist of S1PR1,3,4,5. The first S1PR modulator approved for the treatment of relapsing MS was fingolimod (Currò et al., 2017; Peyrin-Biroulet et al., 2017a).

Various preclinical studies have demonstrated its efficacy at ameliorating colitis in animal models of IBD. Treatment of IL-10 knockout mice for 4 weeks efficiently reduced the number of CD4+ T cells in the colonic lamina propria and decreased the production of IFN-γ in the colon (Mizushima et al., 2004; Huwiler and Zangemeister-Wittke, 2018). Similar data were reported with other colitis models, such as dextran sulfate sodium (DSS), trinitrobenzene sulfonic acid, and T cell transfer into immunocompromised mice (Deguchi et al., 2006; Daniel et al., 2007; Radi et al., 2011; Montrose et al., 2013; Huwiler and Zangemeister-Wittke, 2018). The clinical use of fingolimod in IBD has not been tested, and other, more selective, S1PR modulators are being developed for clinical use in IBD (Currò et al., 2017).

**KRP-203** (Novartis^TM^) is a S1PR1,4,5 agonist and partial agonist of S1PR3. The safety, tolerance, and efficacy of KRP203 were tested in 27 patients with active moderate UC, in a multicenter, double-blind, placebo-controlled study (Radeke et al., 2016; Perez-Jeldres et al., 2018). KRP203 demonstrated adequate tolerance and safety. While KRP203 was shown to be minimally effective with regards to the clinically relevant threshold (novel Bayesian trial design), a 14% in the KRP203 group achieved clinical remission in comparison a 0% in the placebo group (Radeke et al., 2016; Perez-Jeldres et al., 2018). Based on the results of this small study, further development of KRP-203 for ulcerative colitis (UC) was terminated (ClinicalTrials.gov, ClinicalTrials, 2018a).

**Ozanimod**/RPC1063 (Celgene^TM^) is a S1PR agonist, with enhanced selectivity for S1PR1 and S1PR5 (Perez-Jeldres et al., 2018). Ozanimod is metabolized in humans to form one major active metabolite (CC-112273) and other minor active metabolites (RP101988 and RP101075). CC-112273 is responsible for much of the total activity of ozanimod in human with similar potency and selectivity to ozanimod to S1P1 and S1P5 (Scott et al., 2016). Ozanimod is eliminated primarily via biotransformation, followed by biliary excretion. Renal excretion is limited (Hemperly et al., 2018). Its half-life is 19 h, thus upon drug discontinuation, lymphocyte counts return to normal within 72 h (Scott et al., 2016; Hemperly et al., 2018; seekingalpha.com, 2018). However, new data show that the effect could be prolonged by the metabolite CC112273, which has a long 10–13 day half-life, reducing the competitive advantage of ozanimod on the key safety feature of the lymphocyte recovery profile (Scott et al., 2016; Hemperly et al., 2018; seekingalpha.com, 2018). Currently, there is an ongoing phase 1, randomized, parallel-group, open-label study to evaluate the effect of the modulators of the cytochrome P450 (CYP) 2C8 and/or 3A on the single-dose pharmacokinetics of ozanimod and CC112273 in healthy adult subjects (Scott et al., 2016; ClinicalTrials.gov, ClinicalTrials, 2018r; seekingalpha.com, 2018). The TOUCHSTONE phase 2 trial randomized 197 adults with moderate-to-severe UC to either ozanimod 0.5 or 1 mg or placebo daily for up to 32 weeks (Sandborn et al., 2016; White et al., 2018). After 8 weeks induction, 13.8 and 16.4% of patients (0.5 and 1 mg, respectively) reported clinical remission, versus 6.2% in the placebo group. At the same time, clinical response rates were achieved in 57, 54, and 37% for 1, 0.5, and placebo groups, respectively. Mucosal improvement/healing was observed in approximately 30% of the patients treated in each dose group in comparison with 12% in the placebo group. Moreover, at 32 weeks there was an observed improvement in the rates of clinical remission (21, 26, and 6% for 1, 0.5, and placebo, respectively), and 51% of the patients treated with 1 mg had a clinical response, versus 35 and 20% in the groups treated with 0.5 mg and placebo, respectively. Mucosal improvement/healing did not show major differences in comparison with 8 weeks. Ozanimod treatment (1 mg/kg dose) was associated with a greater proportion of histological remission (defined as a Geboes score ≤ 2) at both 8 and 32 weeks (Sandborn et al., 2016). The long-term follow-up of patients involved in TOUCHSTONE study demonstrated that ozanimod was safe, effective, and well tolerated (Sandborn et al., 2016; White et al., 2018).

Initial results of a phase 2, open-label study in 69 patients treated with ozanimod for moderate-to-severe CD demonstrated a meaningful clinical improvement at week 4 and endoscopic improvement at week 12 (Feagan et al., 2017). Phase 3 studies investigating the role of ozanimod in IBD are in progress (White et al., 2018).

**Etrasimod**/APD334 (Arena^TM^) is a S1PR1,4,5 selective agonist. Preliminary data from phase 2 OASIS trial in moderate-to-severe UC were reported (prnewswire.com, 2018). The primary objective, defined as an improvement in 3-component Mayo Clinic Score (stool frequency, rectal bleeding, and endoscopy), was met at 12 weeks. In addition, the 2 mg group achieved significant endoscopic improvement compared with placebo (41.8 versus 17.8%), and also the clinical remission was significant in the 2 mg group compared with placebo (24.5 versus 6.0%). Etrasimod was well tolerated and few patients had serious adverse events (SAEs). Arena plans to start a Phase 3 trial for UC (prnewswire.com, 2018).

**Amiselimod/**MT-1303 (Mitsubishi Tanabe Pharma Corporation^TM^) is an oral selective S1PR1,5 receptor developed for the therapy of autoimmune/immune-mediated disorders. The efficacy and safety of MT-1303 were studied in a phase 2 trial in CD, but the results have not yet been published (ClinicalTrials.gov, ClinicalTrials, 2018c,d). Amiselimod was also investigated for UC, MS, and other immune-mediated diseases; however, its development was discontinued (Peyrin-Biroulet et al., 2017a).

### Safety and Adverse Events

#### Infections

In general, S1PR modulators maintain immune surveillance against pathogens because their effects on effector memory T cell traffic are limited (Perez-Jeldres et al., 2018). However, serious infections, such as disseminated varicella zoster and herpes are rare, but have been reported with fingolimod (Pelletier and Hafler, 2012). In post-marketing surveillance studies, there have been cases of progressive multifocal leukoencephalopathy (PML) and cryptococcal meningitis with the treatment of fingolimod, and without previous use of natalizumab (Olivera et al., 2016; fda.gov, 2018). However, it is necessary to emphasize that the risk to develop PML is low with fingolimod in the absence of prior natalizumab treatment. It is estimated that the risk with fingolimod use is less 1:10,000 patients. The Novartis safety database has identified 15 cases of PML with the use of fingolimod in monotherapy, as of August 2017 (Berger et al., 2018).

#### Cardiovascular Events

Reported cardiovascular events include transient bradycardia, atrioventricular block, and hypertension with the fingolimod use (Olivera et al., 2016; Sandborn et al., 2016). These side effects are attributed to S1PR2 and S1PR3 modulation. The development of selective S1PR1 modulators could theoretically bypass these side effects. However, S1PR1 is found in atrial cardiomyocytes, leading to a dose-dependent reduction in heart rate. In the TOUCHSTONE trial, a patient with preexisting bradycardia developed an asymptomatic, transient bradycardia, and first-degree AV block. The episode was self-limited without the need for treatment. These side effects could be minimized with a gradual dose titration regimen (Olivera et al., 2016; Sandborn et al., 2016).

#### Malignancy

Isolated cases of breast and skin cancer have been identified (Pelletier and Hafler, 2012). Squamous-cell carcinoma of the skin was reported in the TOUCHSTONE trial in one patient on 1 mg of ozanimod. This patient had also received mercaptopurine for more than 2 years (Sandborn et al., 2016).

### Leukopenia

A dose-dependent and sustained decrease in lymphocyte count has been reported, consistent with the drug’s MOA. However, it is reversible with drug discontinuation (Peyrin-Biroulet et al., 2017b).

#### Pregnancy

The teratogenicity risk of this group of drugs is unknown, so it is not recommended for use during pregnancy (Scott et al., 2016).

#### Others Adverse Events

Pulmonary disorders, elevated liver enzymes, and macular edema have been reported (Olivera et al., 2016).

### Future Directions for the S1P Pathway

Ponesimod, Ceralifimod, Siponimod AUY954, SEW2871, AUY954, W061, CS-0777, and GSK2018682 are currently being investigated for use in other autoimmune/immune-mediated disorders (Park and Im, 2017). The pathways involved in the synthesis, degradation, and the mechanism of transport of these molecules represent an attractive new area of research (Perez-Jeldres et al., 2018).

#### Sphingosine Kinases

There are two isoforms of sphingosine kinase (SphK), SphK1 and SphK2. TNF induces SphK1 activation, leading to cyclooxygensase-2 (COX-2) expression and production of prostaglandin E2 (PGE2) that may contribute to mucosal inflammation (Pettus et al., 2003). Moreover, SphK1 expression was found to be elevated in both colonic epithelial cells and inflammatory cells in patients with UC patients correlating with COX2 overexpression (Snider et al., 2009). Data from mice indicate that the SphK1/S1P pathway participates in the development and maintenance of intestinal inflammation (Snider et al., 2009; Wollny et al., 2017). Thus, inhibition of this enzyme could represent a potential new target.

#### Sphingosine Phosphatase

This enzyme, expressed in the gastrointestinal tract, catalyzes dephosphorylation of S1P to sphingosine, resulting in regulation of S1P levels. Elevated sphingosine phosphatase expression has been demonstrated in colitis and contributes to its pathogenesis by disrupting barrier integrity, indicating that its inhibition may have beneficial effects in IBD (Huang et al., 2016).

#### S1P Lyase

Sphingosine-1-phosphate lyase degrades S1P irreversibly. This enzyme is abundant in tissues (Wollny et al., 2017), maintaining low levels S1P in the colonic mucosa in relation with the blood. This favors lymphocyte recirculation from the intestine back into circulation. Its inhibition may impair intestinal lymphocyte egress, but its effect still remains unclear with evidence that shows amelioration of DSS colitis, while other studies show worsening disease (Degagné et al., 2014; Shirakabe et al., 2018).

#### Spinster Homolog 2

The expression of this intra- and extracellular S1P transporter is upregulated in patients with IBD. Thus, it may represent another way to regulate S1P levels for therapeutic purposes (Miklossy et al., 2013).

### Positioning of Small Molecules in the Therapeutic Algorithm of IBD

The choice of IBD treatment must be personalized according to the activity, severity, phenotype, preferences of the patients, comorbidities, history of the therapies used previously, and surgery (Kornbluth and Sachar, 2010; Panes and Alfaro, 2017; Lichtenstein et al., 2018).

The current treatment for IBD is based on aminosalicylates, steroids, immunosuppressants, and biologic therapies (Kornbluth and Sachar, 2010; Panes and Alfaro, 2017; Lichtenstein et al., 2018). The 5-ASA compounds are used as first line in mild-to-moderate UC, and in some cases of IBD-associated arthritis (sulfasalazine). These drugs have an excellent safety profile. Immunosuppressants can be added during maintenance therapy in cases of moderate severity, or in combination with biologic therapy in moderate-to-severe cases due to their synergism or to decrease the immunogenicity of the biologic (Kornbluth and Sachar, 2010; Panes and Alfaro, 2017; Lichtenstein et al., 2018). In recent years, measuring drug and antibody levels has allowed optimization of biological therapies and assisted in avoiding misuse of biologics by under dosing or drug failure (absence of response despite adequate drug level) (Kornbluth and Sachar, 2010; Panes and Alfaro, 2017; Lichtenstein et al., 2018). The calcineurin inhibitors have a limited role in the treatment due their narrow therapeutic window and side effects. Thus, they are mostly being used as a bridge to another maintenance drug in cases of acute severe colitis refractory to corticosteroid. However, in this last case infliximab seems to be a better option, due to less toxicity in comparison with cyclosporine (Kornbluth and Sachar, 2010; Panes and Alfaro, 2017; Lichtenstein et al., 2018).

New SM offers an alternative to the current therapeutic arsenal, especially in cases of steroid-resistance and cases of nonresponse and/or are intolerance to conventional therapies.

Precise positioning the new small drugs in the therapeutic armament for IBD is difficult in the absence of head-to-head randomized controlled trials. The SM have a role in the treatment of moderate-to-severe IBD due to the lack of immunogenicity, and potential intermittent “on-off” dosing without resultant antibody formation and loss of response.

Most information available is for tofacitinib, approved for UC moderate-to-severe active, being a good option in cases refractory to anti-TNFa. Its effectiveness in comparison with anti-TNF as first-line therapy in moderate-to-severe UC, their use as combination therapy for example with other drugs as vedolizumab, its sequential use with other drugs (for example, induction with tofacitinib, followed by vedolizumab), or even it uses in acute severe colitis refractory to steroids, must also be evaluated in clinical trials, before authoritative consensus recommendations. In the absence of head-to-head comparisons, the evidence favors the use of infliximab in hospitalized patients with acute severe colitis in perianal disease.

Furthermore, it is important to consider tofacitinib’s safety profile and may be premature recommend its use in combination with immunomodulators, anti-TNFa, and/or cyclosporine, until additional safety information is available.

Improved knowledge of the mechanisms regulating disease, by genome sequencing analysis, improved comprehension of the immunological pathways, and further understanding of the role of the microbiome, may lead to new targets. In fact, it is possible that future therapies will be chosen not only by considering traditional patient characteristics, but also based on the patient’s microbiome and immune genotype, as well as predictive modeling of drug responses validated prospectively.

## Conclusion

Novel, orally available drugs represent a new and exciting option in the IBD therapeutic arsenal, showing efficacy and reasonable safety. However, more studies are required to define their safety related to infection, malignancy, and pregnancy. One of the clear advantages of SMs is their lack of immunogenicity and their short half-life which represents an advantage when adverse events may mandate interruption of therapy. Other advantages include the administration route, maintenance of T cell effector memory response, potentially lower manufacturing cost, and finally, the new agents are more receptor-specific (Perez-Jeldres et al., 2018). The positioning of these new drugs with relation to existing treatment paradigm remains uncertain.

## Author Contributions

JR-N, TP-J, WS conception of work. JR-N, TP-J design of work. JR-N, TP-J drafting of manuscript. JR-N, WS, DP, AY, DG, JB, TK, CT, SY, and LL critical revision of manuscript. JR-N, TP-J, WS, DP, AY, JB, TK, CT, DG, SY, and LL final approval of work. JR-N, TP-J, WS, DP, AY, JB, TK, CT, DG, LL,and SY agreement to be accountable for all aspects of presented work.

## Conflict of Interest Statement

WS reports research grants from Atlantic Healthcare Limited, Amgen, Genentech, Gilead Sciences, Abbvie, Janssen, Takeda, Lilly, Celgene/Receptos; consulting fees from Abbvie, Allergan, Amgen, Boehringer Ingelheim, Celgene, Conatus, Cosmo, Escalier Biosciences, Ferring, Genentech, Gilead, Gossamer Bio, Janssen, Lilly, Miraca Life Sciences, Nivalis Therapeutics, Novartis Nutrition Science Partners, Oppilan Pharma, Otsuka, Paul Hastings, Pfizer, Precision IBD, Progenity, Prometheus Laboratories, Ritter Pharmaceuticals, Robarts Clinical Trials (owned by Health Academic Research Trust or HART), Salix, Shire, Seres Therapeutics, Sigmoid Biotechnologies, Takeda, Tigenix, Tillotts Pharma, UCB Pharma, Vivelix; and stock options from Ritter Pharmaceuticals, Oppilan Pharma, Escalier Biosciences, Gossamer Bio, Precision IBD, Progenity. AJ-R is consultant and part of the speaker bureau for Prometheus Laboratories and employee of Prometheus Laboratories. DP has been a speaker bureau ABBVIE. The remaining authors declare that the research was conducted in the absence of any commercial or financial relationships that could be construed as a potential conflict of interest.
